# Adsorption Characteristics and Charge Transfer Kinetics of Fluoride in Water by Different Adsorbents

**DOI:** 10.3389/fchem.2022.917511

**Published:** 2022-06-16

**Authors:** Jiaxi Tang, Biao Xiang, Yu Li, Ting Tan, Yongle Zhu

**Affiliations:** ^1^ College of Environmental Science and Engineering, Liaoning Technical University, Fuxin, China; ^2^ Liaoning Academy of Agricultural Sciences, Shenyang, China

**Keywords:** fluoride, electron binding, adsorption, kinetics, charge transfer

## Abstract

Water containing high concentrations of fluoride is widely distributed and seriously harmful, largely because long-term exposure to fluoride exceeding the recommended level will lead to fluorosis of teeth and bones. Therefore, it is imperative to develop cost-effective and environmentally friendly adsorbents to remove fluoride from polluted water sources. In this study, diatomite (DA), calcium bentonite (CB), bamboo charcoal (BC), and rice husk biochar (RHB) were tested as adsorbents to adsorb fluoride (F^‐^) from water, and this process was characterized by scanning electron microscopy (FEI-SEM), X-ray diffraction (XRD), and Fourier-transform infrared spectroscopy (FT-IR). The effects of pH, dosage, and the initial mass concentration of each treatment solution upon adsorption of F^‐^ were determined. Kinetic and thermodynamic models were applied to reveal the mechanism of defluoridation, and an orthogonal experiment was designed to obtain the optimal combination of conditions. The results show that the surfaces of CB, BC, and RHB have an irregular pore structure and rough surface, whereas DA has a rich pore structure, clear pores, large specific surface area, and high silica content. With regard to the adsorption process for F^‐^, DA has an adsorption complex electron interaction; that of CB, BC, and RHB occur mainly *via* ion exchange with positive and negative charges; and CB on F^‐^ relies on chemical electron bonding adsorption. The maximum adsorption capacity of DA can reach 32.20 mg/g. When the mass concentration of fluoride is 100 mg/L, the pH value is 6.0 and the dosage is 4.0 g/L; the adsorption rate of F^‐^ by DA can reach 91.8%. Therefore, we conclude that DA soil could be used as an efficient, inexpensive, and environmentally friendly adsorbent for fluoride removal, perhaps providing an empirical basis for improving the treatment of fluorine-containing water in the future.

## 1 Introduction

Fluoride is widely distributed in nature, being one of the abundant trace elements in the Earth’s crust, where its content is 625 mg/g (Amini et al., 2008; Ozsvath, 2009). Fluoride, as a pollutant in water, is produced not only from natural geological resources but also from industries that use fluorine-containing compounds as raw materials (Xu et al., 2015). However, for the human body, fluorine is an essential micronutrient and one of the main components of human teeth and bones. Thus, an appropriate intake of fluorine can prevent dental caries and improve bone strength. However, excessive intake of fluoride will cause harmful health effects, such as tooth and bone fluorosis and impaired fetal brain functioning (He X. et al., 2020). The concentration level of F^‐^ in drinking water should be lower than the 1.5 mg/L, as stipulated by the World Health Organization (WHO) (Wang et al., 2013). Currently, endemic fluorosis has become a very serious health problem worldwide, and millions of people are affected by high concentrations of fluoridated drinking water. Therefore, it is necessary to develop fluoride-reduction technologies, especially those that are low-cost, efficient, and environmentally friendly.

Many experts and scholars in related fields have conducted extensive research on techniques for the removal and recovery of fluoride from water bodies. The common fluoride removal methods include chemical precipitation (Liu and Liu, 2016), reverse osmosis (Ndiaye et al., 2005), ion exchange (Pintar et al., 2001), nanofiltration (Shen and Schafer, 2014), and adsorption (Paudyal et al., 2011; Jagtap et al., 2012), among others, of which adsorption is most commonly used (He J. et al., 2020). Adsorption is the mass transfer process of adsorbing gas or solute onto the surface of a solid or liquid. When the molecules or atoms on the solid surface have residual surface energy due to force imbalance, the process of adsorption on the solid surface will occur to attract and maintain the colliding material (Ghosh et al., 2021). Currently, those adsorbents that can remove water pollutants mainly include carbon-based adsorbents, nanoadsorbents, metal oxides, hydroxide-based adsorbents, resins, and modified and composite adsorbents (Igwegbe et al., 2018). Yet, the application of many adsorption materials entails advantages and disadvantages. Nevertheless, their low cost and efficiency are seen as their chief advantages. Low-cost and widely used adsorbents include diatomite (Yitbarek et al., 2019), bentonite (Ortiz-Ramos et al., 2022), and biochar (Mei et al., 2020), all of which have great application value for fluoride’s removal from water. Diatomite is a light-colored, soft, and lightweight sedimentary rock composed of amorphous silica (SiO_2_-nH_2_O), mainly derived from aquatic diatom plant skeletons. Due to its high porosity, large surface area, high permeability, low density, small particle size, heat resistance, and chemical stability (Hadjar et al., 2008; Zhang et al., 2013), it has been used for removing different pollutants in various water bodies. Xu et al. (2015) used natural diatomaceous earth for the purification of high fluoride wastewater and found that at a pH of 5 and a dosing rate of 50 g/L, 82% of fluoride was adsorbed. Similar to diatomite, bentonite is a clay mineral, whose main component is montmorillonite, which has a large surface area and strong adsorption capacity and is abundant in nature. Studies on the use of bentonite or modified bentonite for fluoride removal from water bodies have also been reported, for which good adsorption results are reported (Fang et al., 2016; Kalsido et al., 2021). Biochar is a typical carbon-based adsorbent obtained by high-temperature pyrolysis of waste biomass in an anaerobic environment. Biochar has a well-developed pore structure, a high specific surface area, excellent ion exchange properties, abundant surface functional groups, and good stability (Wang et al., 2019). Some studies have shown that biochar prepared using rice husk is capable of up to 72% adsorption of fluoride from water bodies (Pillai et al., 2020).

In summary, many studies have examined the use of adsorption materials to remove fluoride in water, but the adsorption capacity and action mechanism of different adsorption materials are likely to differ. Accordingly, in this study, diatomite (DA), calcium bentonite (CB), bamboo biochar (BC), and rice husk biochar (RHB) were selected to systematically study their adsorption properties for fluoride (F^‐^) in water and to screen for material’s best enabling adsorption for fluoride. A second objective was to investigate the adsorption mechanism for fluoride in water in high-fluorine areas to provide a theoretical basis for the treatment of fluoride-containing water.

## 2 Materials and Methods

### 2.1 Experimental Materials and Characterization

The DA used in the experiments was supplied from the Jiangsu Chengbo Environmental Protection Technology Co., Ltd., China, with an average particle size of 9.14 μm. BC and RHB were purchased from the Lize Environmental Technology Co., Ltd., Zhengzhou City, Henan Province, China, with average particle sizes of 6.71 and 10.43 μm, respectively. CB was purchased from Fuxin General Building Materials Factory, China, with an average particle size of 6.76 μm.

The microscopic morphology and surface characteristics of the four adsorbent materials were observed by ultra-high resolution field emission scanning electron microscopy (FEI-SEM, FEI-Verios 460L). Their crystal structures were characterized by X-ray diffraction (XRD, Ultima IV, Nippon Science), and their surface structural groups were analyzed by Fourier transform infrared spectroscopy (FT-IR, IS50, Thermofisher).

### 2.2 Experimental Reagents and Apparatus

Sodium fluoride (GR) was used (from the Tianjin FengChuan Chemical Reagent Technology Co., Ltd., China)to simulate aqueous fluoride (F^‐^). The experimental water was deionized; the buffer solutions used for the experiment were sodium citrate dihydrate and sodium nitrate (respectively from the Tianjin Guangfu Technology Development Co., Ltd. and Shenyang Huadong Reagent Factory, China). The instruments used in the experiment were a JA1003 electronic balance, a PHS-3C type pH meter, a BS-MS thermostatic oscillator, an L550 Xiang Yi centrifuge, a CM-230 laboratory pure water treatment system, a PXS-270 fluorometer, and a JB-10 magnetic stirrer.

### 2.3 Adsorption Experiments

#### 2.3.1 Effect of the Initial Mass Concentration of F^‐^ on the Adsorption Effect

The initial mass concentration gradient of F^‐^ was adjusted to 0, 10, 20, 40, 60, 80, 100, and 150 mg/L, at pH 6.0, and 25 ml per concentration was measured in 50 ml centrifuge tubes. To these, 0.10 g of each of the four adsorbent materials was added (separately), and the adsorption experiment was carried out at 25°C for 120 min in a constant temperature shaker at 200 rpm. After centrifugation at 4,000 r/min for 10 min, 10 ml of the supernatant was centrifuged through a 0.45 μm filter membrane, and the remaining F^‐^ mass concentration in the solution was determined using a PXS-270 fluoride ion-selective electrode. Each adsorbent was tested for each F^‐^ concentration in triplicate, and relative standard deviations of duplicate samples were less than 5.0%.

#### 2.3.2 Adsorption Kinetics Experiments

The kinetic parameters of F^‐^ (100 mg/L) sorption by each adsorbent (0.10 g) were determined with 25 ml solutions for 5, 10, 20, 40, 60, 120, 240, 360, 480, and 720 min at 25 °C in the solution at pH 6. After centrifugation at 4,000 r/min for 10 min, 10 ml of the supernatant was centrifuged through a 0.45 μm filter membrane, and the remaining F^‐^ mass concentration in the solution was measured.

#### 2.3.3 Effect of pH on the Adsorption Effect

, About 25 ml of an F^‐^ solution (100 mg/L) was added into 50 ml centrifuge tubes, and its pH was adjusted to 3.0, 4.0, 5.0, 6.0, 7.0, or 8.0. To each tube, 0.10 g of DA was added, and all tubes were stored at 25°C. The adsorption experiment was the same as given in Section 2.3.1. After centrifugation, 10 ml of the supernatant was taken and passed through a 0.45 μm filter membrane to determine the remaining F^‐^mass concentration in the solution.

#### 2.3.4 Effect of Dosage on the Adsorption Effect

The DA was weighed at 0.04, 0.08, 0.10, 0.15, 0.20, and 0.30 g in 50 ml centrifuge tubes, and 25 ml of a mass concentration of F^‐^ (100 mg/L) in solution was added to each tube. The adsorption experiment was the same as given in Section 2.3.1. After centrifugation at 4,000 r/min for 10 min, 10 ml of the supernatant was centrifuged through a 0.45 μm filter membrane and then the remaining F^‐^ mass concentration in the solution was measured.

#### 2.3.5 Orthogonal Experiments

According to the design method of the orthogonal test, the factors selected for investigation were as follows: adsorption pH (A), the mass concentration of the F^‐^ solution (B), and adsorbent dosage (C) on the adsorption of F^‐^ in water. Three levels of each factor were selected for the three-factor, three-level orthogonal test of L_9_3^4^.

### 2.4 Data Processing

The removal rate is calculated as:

$$R=\frac{\left(\rho_{0}-\rho_{e}\right)}{\rho_{0}}\times 100\%.$$ (1)

where $\rho_{0}-F^{-}$ is the initial mass concentration of the solution (mg/L), $\rho_{e}$ is the mass concentration of F^‐^ in solution at the adsorption equilibrium (mg/L), and A is the adsorption rate.

The adsorption volume is calculated as follows:

$$q_{e}=\frac{\left[\left(\rho_{0}-\rho_{e}\right)\times V\right]}{m}.$$ (2)

where $\rho_{0}-F^{-}$ is the initial mass concentration of the solution (mg/L), $\rho_{e}$ is the mass concentration of F^‐^ in the solution at the adsorption equilibrium (mg/L), V is the volume of the solution (L), m is the material dosage (g/L), and $q_{e}$ is the adsorption amount of F^‐^ at the adsorption equilibrium (mg/g).

The two adsorption fitting models are as follows. The Langmuir model equation is

$$\frac{\rho_{e}}{q_{e}}=\frac{1}{Q_{m}K_{L}}+\frac{\rho_{e}}{Q_{m}}.$$ (3)

The Freundlich model equation is

$$\log q_{e}=\log K_{f}+\frac{1}{n}\log\rho_{e}.$$ (4)

where $\rho_{0}-F^{-}$ is the mass concentration of F^‐^ in the solution at the adsorption equilibrium (mg/L), $q_{e}$ is the adsorption amount of F^‐^ at the adsorption equilibrium (mg/g), $Q_{m}$ is the maximum saturation adsorption amount (mg/g), $K_{L}$ is a constant in Langmuir’s equation regarding the heat of adsorption, and N is a constant in the Freundlich equation related to the adsorption strength, preferential adsorption for *n* > 1, linear adsorption for *n* = 1, and non-preferential adsorption for *n* < 1.

The quasi-first-order kinetic equation is

$$ln\left(q_{e}-q_{t}\right)=lnq_{e}-K_{1}t.$$ (5)

The quasi-second-order kinetic equation is

$$\frac{t}{q_{t}}=\frac{1}{K_{2}q_{e}^{2}}+\frac{t}{q_{e}}.$$ (6)

where $q_{e}$ is the equilibrium adsorption amount (mg/g), $q_{t}$ is the adsorption amount at moment *t* (mg/g), t is the adsorption time (min), $K_{1}$ is the quasi-first-order kinetic adsorption rate constant (min-1), and $K_{2}$ is thequasi-second-order kinetic adsorption rate constant (min-1).

## 3 Results and Discussion

### 3.1 Characterization of the Adsorbent Materials

#### 3.1.1 FEI-SEM Analysis

**FIGURE 1:**
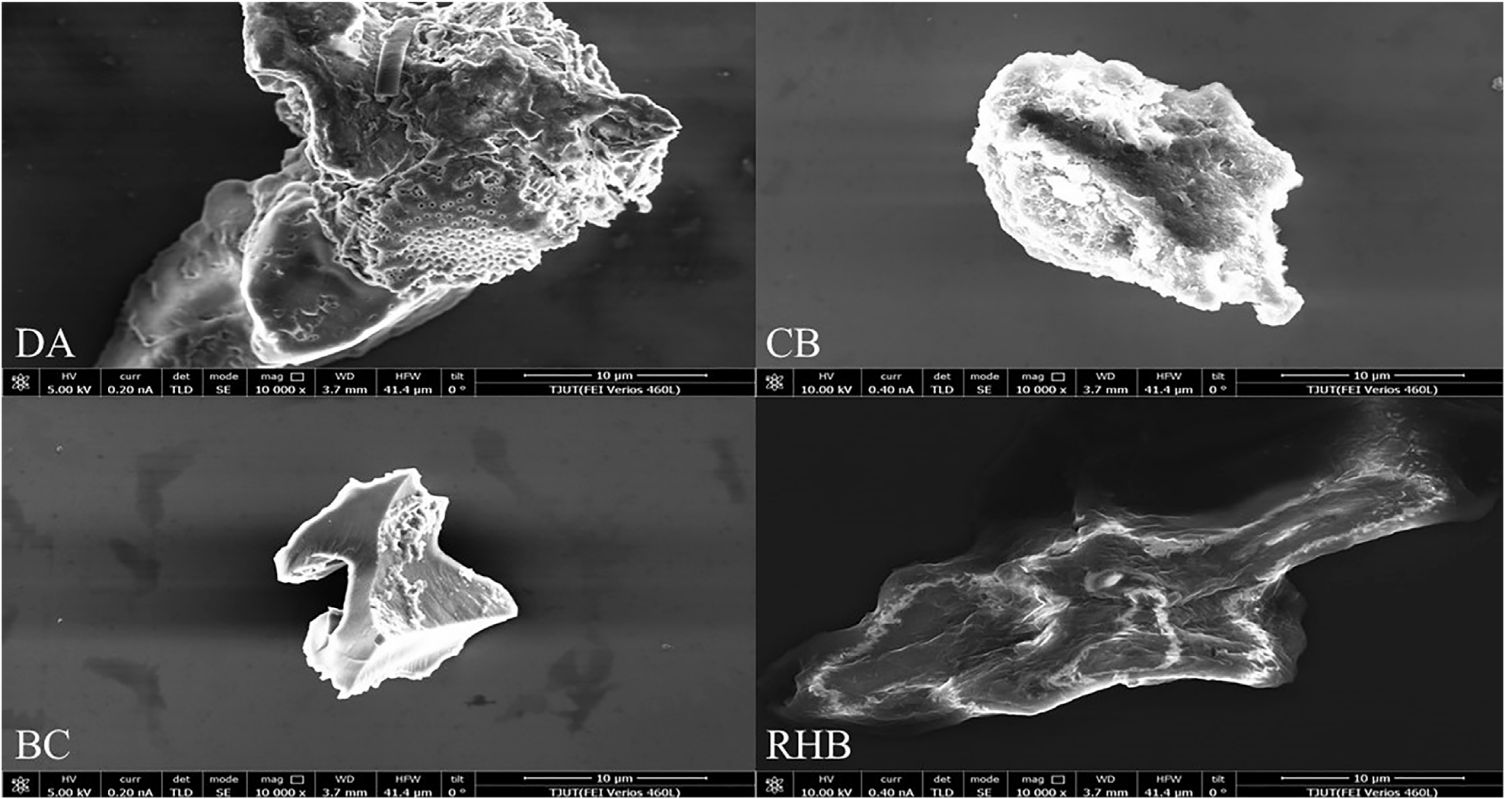
FEI-SEM images of the four adsorbent materials.

Figure 1 shows the scanning electron micrographs of the four materials under a 10,000 × microscope. Evidently, the surface of DA has two types of pore structures with large and small pores and clear pores, indicating that DA has a unique multi-level pore structure. The DA’s main component is silica, which has a large specific surface area and good thermal stability, and is a natural green material water treatment agent with a porous structure (Akafu et al., 2019); therefore, fluoride is likely to be adsorbed on DA. The surface of CB has an irregular pore structure but a rough surface; while for BC and RHB, they have relatively few pores, showing the presence of dispersed particulate matter as well as a plate-like structure. The granular material on their surface could be crystals formed by some mineral elements (Piri et al., 2020). Overall, DA has a relatively well-developed pore structure and a high specific surface area for better absorption.

#### 3.1.2 XRD Analysis

**FIGURE 2:**
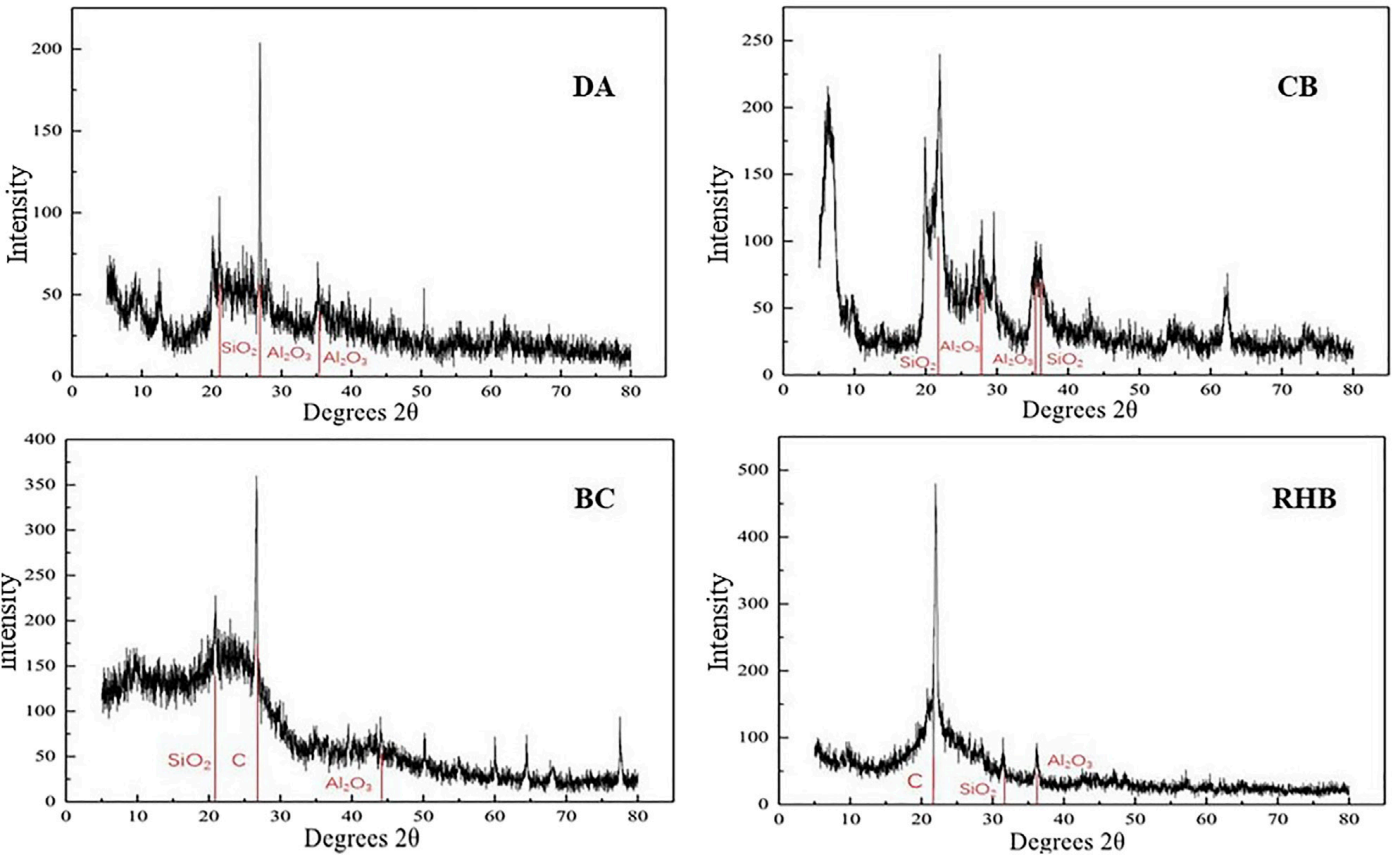
XRD patterns of the four adsorbent materials.

Figure 2 shows the XRD plots of the four materials. Evidently, DA and CB have wider diffraction peaks at around 22.0°, whose main composition corresponds to amorphous SiO_2_. The sharp diffraction peaks at around 26° can be attributed to quartz impurities in the materials, while the wider diffraction peaks at around 35° correspond to amorphous Al_2_O_3_ (Xu et al., 2015). In terms of composition, DA and CB were polycrystalline. For BC and RHB, the main XRD diffraction peaks 2θ of the biochar crystals are at 25° and 27°, respectively (Yihuan et al., 2022). The relatively higher intensity of diffraction peaks for RHB than BC can arise from the better crystallization properties of carbon. In addition, all four materials contain SiO_2_ and Al_2_O_3_, and these metal cations can adsorb F^−^ *via* electrostatic gravity, thus providing adsorption sites on the material surface (Teng et al., 2009). Moreover, the specific surface area and adsorption efficiency of these materials can be significantly increased because they are rich in surface-active functional groups (Cui et al., 2013).

#### 3.1.3 FT-IR Analysis

**FIGURE 3:**
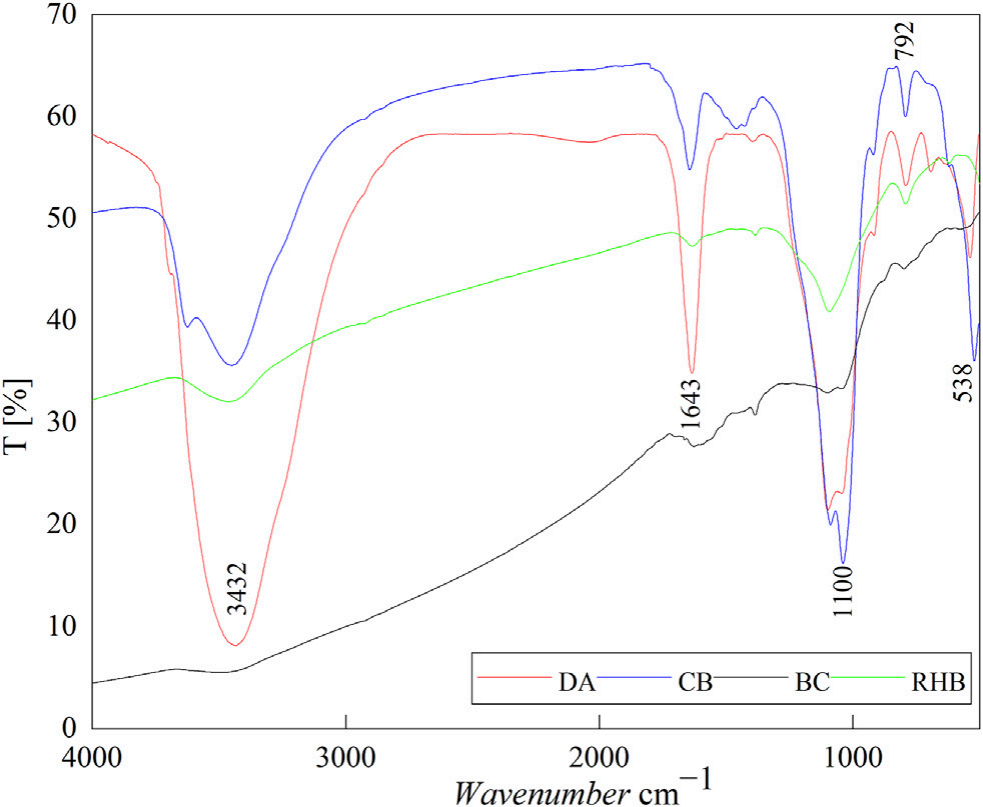
Infrared spectra of the four adsorbent materials.

The four adsorbents were identified in detail using their respective FTIR analysis data (Figure 3). The vibrations of DA, CB, and RHB of about 3,432 cm^−1^ are due to the stretching vibrations of the adsorbed water hydroxyl group (O-H) and the surface hydroxyl group. All four materials have vibrational peaks around 1,634 cm^−1^, probably from the bound water or the surface hydroxyl group. The peaks of DA, CB, BC, and RHB located at 1,100 cm^−1^ and 538 cm^−1^ are of siloxane groups (Si-O-Si-), and the peak at 792 cm^−1^ is attributable to the Al-O absorption band (Akafu et al., 2019). These adsorbent materials are rich in oxygen-containing functional groups and thus can provide Π-electrons for surface complexation with F^‐^, resulting in stable structures (Datsko and Zelentsov, 2016). Additional studies have shown that silanol groups are very active and can react with many contaminants, including fluoride, through the formation of hydrogen bonds (Al-Ghouti et al., 2003). The formation of new bonding electronic structures by complexation with the F^‐^ is one of the main mechanisms by which fluoride ions are adsorbed, as seen in the infrared spectra.

### 3.2 Effect of Different F^‐^ Mass Concentrations on the Adsorption Effect

**FIGURE 4:**
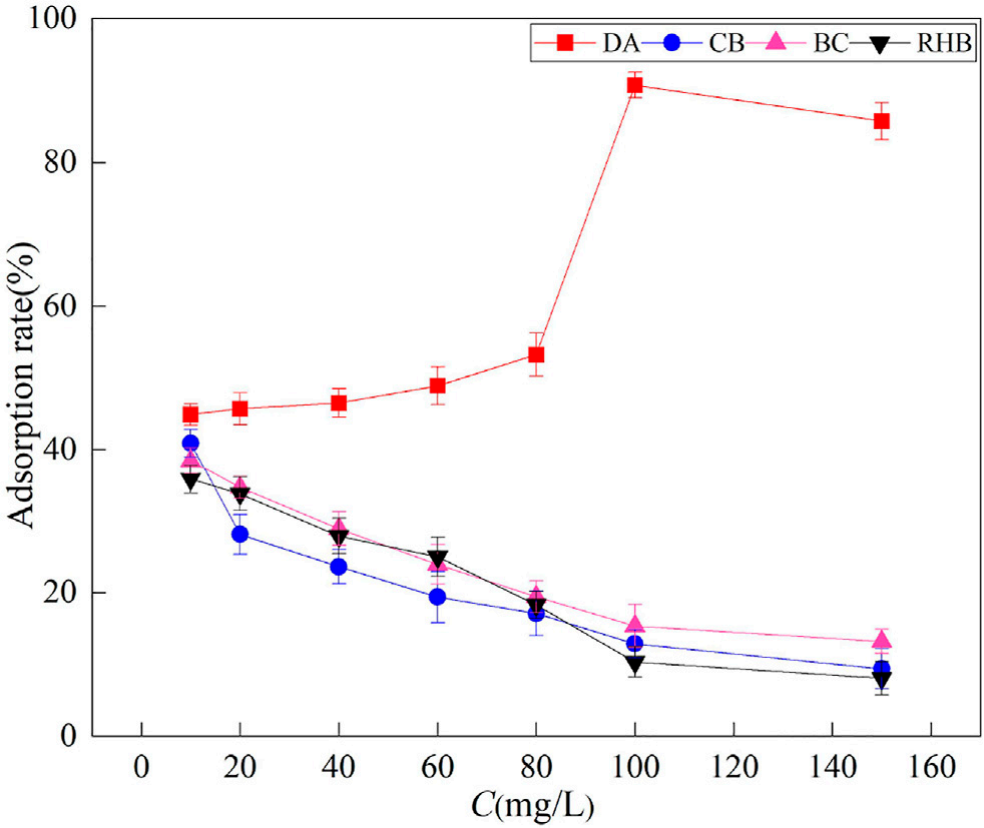
Effects of four adsorbent materials on the adsorption of an F^‐^ solution with different mass concentrations.

The adsorption rate of F^‐^ by DA increased with an increasing initial mass concentration of F^‐^ (Figure 4). The highest adsorption rate of 90.7% was achieved when the mass concentration was 100 mg/L. This is because at low concentrations, DA has a sufficient number of active sites, and therefore, most of the F^‐^ interacts with the active sites on DA, leading to greater adsorption of F^‐^ (Akafu et al., 2019). When the concentration reaches a certain value, the adsorption rate of F^‐^ by DA begins to decline. This is because at higher concentrations, the active sites of the adsorbent are saturated and fluoride ions outnumber the adsorption sites, and at a constant mass of the adsorbent, the ratio of F^‐^ to the available active surface sites is higher with an increasing initial F^‐^ concentration, leading to a declining adsorption rate (Bhaumik et al., 2013). For the other three materials, their adsorption rates decreased with an increasing initial mass concentration of F^‐^ because the adsorption sites of the three materials were saturated at this concentration; hence, the adsorption rate decreased as the concentration increased. The maximum adsorption rate of CB (41%) was higher than that of BC and RHB. It can be concluded that the magnitude of adsorption performance of the four materials is DA > CB > BC > RHB. Gomoro et al. (2012) showed that the efficiency of fluoride adsorption by adsorbents decreases as the initial mass concentration of fluoride increases and reaches a certain threshold value. This finding agrees with our results. Hence, the effectiveness of electron exchange at the active sites is the main factor determining the adsorption of fluoride ions.

The Freundlich and Langmuir models were used here to describe the adsorption of F^‐^ processes of the four materials, and their fitted parameters are in Table 1. The Langmuir adsorption model describes the adsorption of a single molecular layer, the adsorption mechanism is mainly ion exchange, and the adsorption is mainly chemisorption, while the Freundlich adsorption model describes a non-homogeneous adsorption behavior, which refers to the adsorption process of multiple molecular layers occurring on the surface and spatially inhomogeneous distribution of the adsorbent, and the presumed adsorption mechanism is mainly adsorption–-complexation interactions (Tang et al., 2020). As shown in Table 1, for DA the Freundlich model fits better than the Langmuir model, with *R* ^2^ values of up to 0.9930, indicating that the adsorption process of DA for F^‐^ is an adsorption–complex electron interaction, surface adsorption, and multi-molecular layer adsorption. When applied to CB and BC, both models fit better, indicating that the adsorption process of F^‐^ by CB and BC is unilamellar and multilamellar co-adsorption, mainly based on ion exchange involving positive and negative charges. For RHB, the Langmuir model fits better than the Freundlich model, with an *R* ^2^ value of 0.9128, indicating that the adsorption process of F^‐^ by RHB is unilamellar. Its adsorption mechanism is mainly based on ion exchange involving positive and negative charges. Similar results were reported by Goswami and Kumar (2018), who studied the adsorption of fluoride using rice husk biochar.

**TABLE 1:** Adsorption fitting parameters of four different tested materials.

Item	Freundlich model			Langmuir model		
	Log *K*_*f*_	*N*	*R* ^2^	*K*_L_	*Q*_*m*_	*R* ^2^
DA	0.717	1.15	0.9930	0.00759	32.20	0.8867
CB	0.975	0.52	0.9483	0.0289	4.42	0.9841
BC	-0.549	1.70	0.9594	0.0177	6.58	0.9764
RHB	-0.384	2.20	0.7201	0.0701	3.42	0.9123

### 3.3 Analysis of Adsorption Kinetics

**FIGURE 5:**
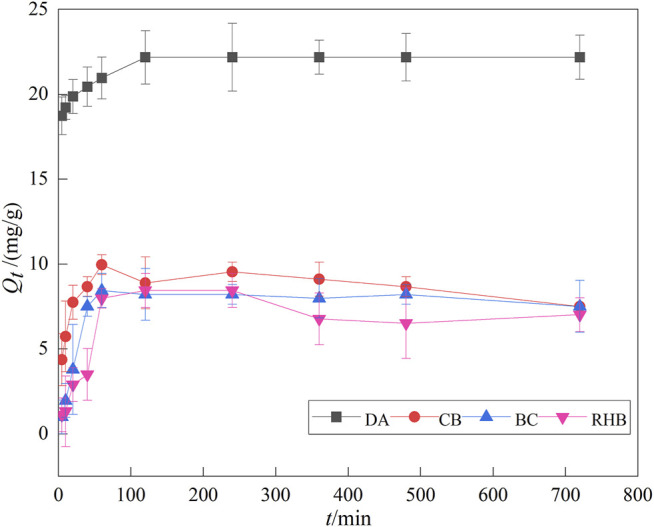
Effect of adsorption time on F^‐^ adsorption.

Figure 5 shows the variation in the adsorption amount of F^‐^ by the four materials over time. Clearly, the adsorption amount increased with the elapsed time, and the maximum adsorption amount was reached at around 120 min. The materials’ adsorption amounts were ranked as follows: DA > CB > BC > RHB; the maximum adsorption amount of DA reached 22.300 mg/g, twice as much as that found for the other three materials. Beyond 120 min, the adsorption amount did not change and adsorption had reached equilibrium. Oladoja and Helmreich (2016) studied the adsorption of fluoride using calcium aluminate–diatomaceous earth composites and found that its adsorption reached equilibrium when the reaction time was 120 min, as found in our study. This is because at the initial stage of adsorption, there are more active sites on the adsorbent surface and so F^‐^ rapidly occupies the active sites on the adsorbent’s surface, and its adsorption capacity increases (Sadhu et al., 2021). But when prolonged, the available active sites on the adsorbent’s surface decline significantly, and adsorption gradually spreads to the internal surface of the porous adsorbent, which strengthens its resistance and slows down adsorption such that the adsorption capacity no longer changes (Akafu et al., 2019).

Two kinetic models, quasi-first-order and quasi-second-order, were used to study the kinetic process of adsorption of F^‐^ by the four adsorbents. The corresponding fitted parameters are shown in Table 2. For DA and CB, the fitting results were better for the quasi-second-order than the quasi-first-order kinetic models, with *R* ^2^ values of 0.9998 and 0.9461, respectively. This suggests that the adsorption process of DA and CB on F^‐^ is dominated by chemisorption, with surface adsorption and physical adsorption acting in concert (Lu et al., 2012). The adsorption rate is mainly controlled by chemisorption, while the adsorption capacity shows a positive correlation with the number of active sites on the adsorbent surface, and the adsorption reaction chiefly occurs *via* the sharing of electrons and the gain and loss of electrons (Aboul-Kassim and Simoneit, 2001). Further, the theoretical adsorption amount obtained by fitting the quasi second-order kinetic equation of DA is closer to the experimental value, indicating that the adsorption of F^‐^ by DA better conforms to quasi second-order kinetics. Oladoja and Helmreich (2014) also confirmed that the adsorption of fluorine by diatomaceous earth is better described by quasi-second-order kinetics. For RHB, in contrast, the fitted quasi-first-order kinetic model outperformed the second-order kinetic model, with an *R* ^2^ value of 0.9527. This indicates that the adsorption of F^‐^ by RHB relies primarily on a physical process. Other studies have shown that due to excess sodium fluoride in the solution, the resulting aluminum fluoride binds NaF to form a NaAlF_4_ intermediate compound, which is transformed into cryolite due to further adsorption of NaF (Datsko and Zelentsov, 2016). The reactions between fluoride ions and their adsorbent are as follows:

$$A1^{3+}+3F^{-}\to A1F_{3},$$

$$A1F_{3}+NaF\to NaA1F_{4},$$

$$NaA1F_{4}+2NaF\to Na_{3}A1F_{6}.$$

**TABLE 2:** Kinetic fitting parameters of four different materials for F^‐^ adsorption.

Item	Quasi-first-order kinetic model			Quasi-second-order kinetic model		
	*q*_*e*_	*K*_1_	*R* ^2^	*q*_*e*_	*K*_2_	*R* ^2^
DA	3.600	0.0182	0.9880	22.300	0.01990	0.9998
CB	0.988	0.0410	0.9234	1.142	0.1644	0.9461
BC	0.913	0.0450	0.7653	1.230	0.1991	0.8128
RHB	0.924	0.7620	0.9527	0.968	0.1756	0.7632

The aforementioned results showed that DA exhibited good adsorption performance for F^‐^; therefore, it was chosen to investigate how other conditions influence the adsorption of F^‐^.

### 3.4 Effects of Different Factors Influencing the Adsorption of F^‐^

#### 3.4.1 Effect of pH

**FIGURE 6:**
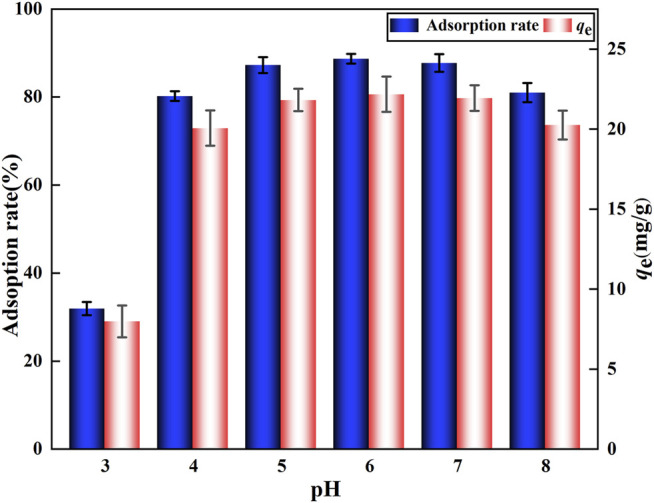
Effect of DA on F^‐^ adsorption at different pH levels.

For the adsorption process, the solution pH is a critical factor that can change the pollutant’s presence and morphology as well as the surface charge of the adsorbent (Zhao et al., 2020). The adsorption rate and adsorption capacity of F^‐^ by DA increased with increasing pH, reaching a maximum at a pH of around 6.0 with an adsorption rate of 88.7% and an adsorption capacity of 22.2 mg/g (Figure 6); however, at a pH > 6.0, the adsorption rate and adsorption capacity of F^‐^ by DA started to decrease, which can be attributed to the competition between hydroxyl ions and fluoride for active adsorption sites (Koilraj and Kannan, 2013). The surface charge of the adsorbent is related to the pH of the surrounding aqueous solution. Neutral or alkaline conditions generally result in a negative charge on the surface. Thus, under acidic conditions, the surface usually retains excess protons, resulting in a total positive charge (Cai et al., 2017). More H^+^ on the adsorbent’s surface leads to the electrostatic attraction between its positively charged surface and F^‐^ under acidic conditions, and the exchange of hydroxyl groups with F^‐^ is considered mainly responsible for the removal of fluoride at the interface with the adsorbent water (Yitbarek et al., 2019). At this time, the adsorption of F^‐^ is favored. A higher pH means more negative charges on the adsorbent surface, which will lead to strong electrostatic repulsion between the adsorbent and F^‐^ as well as a significantly diminished adsorption capacity (Wang et al., 2013). Therefore, weak acid solutions are favorable for DA-driven defluoridation and pH = 6.0 is the optimal adsorption condition for it.

#### 3.4.2 Effect of DA Dosage

**FIGURE 7:**
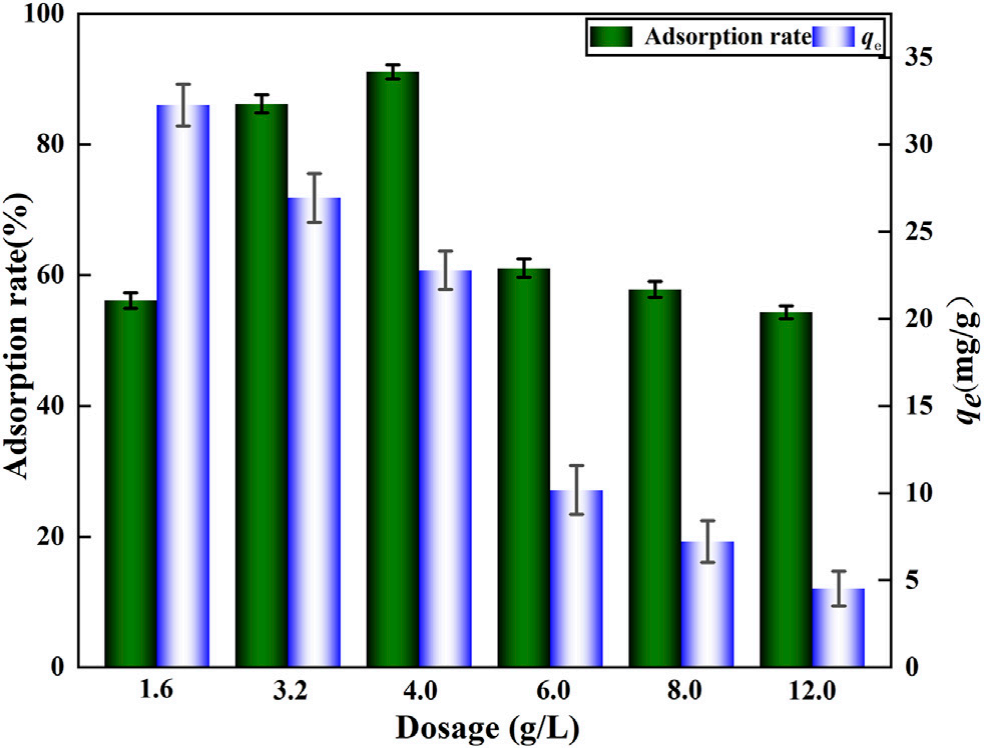
Effects of different dosages of DA on F^‐^ adsorption.

To determine the appropriate amount of DA, adsorption experiments were performed at different DA dosages fixed at pH 6. When the dosage was increased from 1.6 to 4.0 g/L, the F^‐^ adsorption rate increased from 56.1 to 91.1% (Figure 7), but when the dosage was greater than 4.0 g/L, the adsorption rate started to decrease. The increase in adsorption efficiency with a larger adsorbent dose is due to the higher availability of fluoride bound to active surface sites at higher adsorbent doses (Fekadu et al., 2013). Evidently, the adsorption of F^‐^ by DA eventually declines rather with higher dosing, and the weakened adsorption capacity is due to the greater fixed initial fluorine concentration and a solid dose of the fixed solute load, leading to the reduced availability of fluoride ions per unit mass of the adsorbent (Akafu et al., 2019). Given the porous structure of this DA material, it has more fine pores than the other tested adsorbents, likely increasing the specific surface area and contributing to the augmented adsorption capacity of this material. Based on the aforementioned analysis, the suitable amount of added DA should be 4.0 g/L to remove fluoride. Another study pointed out that DA is capable of regeneration after its adsorption of pollutants and that a K_2_SO_4_ solution is better for the regeneration of spent adsorbents (Gitari et al., 2017), thus enabling the recycling and re-use of the material.

### 3.5 Orthogonal Experiments

The DA material with the best effect was selected from orthogonal experiments. The results of the orthogonal experiments are presented in Table 3, namely the values of different influencing factors *K*_1_, *K*_2_, *K*_3_, *k*_1_, *k*_2_, *k*_3_, and their corresponding *R* values. By comparing the *k*_1_, *k*_2_, and *k*_3_ values, the optimal level of each experimental influence factor can be determined, which in turn leads to the optimal combination of experimental conditions. By comparing the differences among *R*-values, the size of the influence of each factor on the experimental results can be determined; a larger *R*-value indicates that the factor has a greater effect on the adsorption process. As seen in Table 3, *R*_A_ > *R*_C_ > *R*_B_, indicating that the effect size of each influencing factor is as follows: dosage > mass concentration > pH. Hence, the optimal combination derived from the experiments was a dosage of 4.0 g/L, pH of 6.0, and a mass concentration of 100 mg/L, under which the adsorption rate of F^‐^ by DA could reach 91.8%.

**TABLE 3:** Orthogonal experimental results for F^‐^ adsorption on DA.

Items	A, dosage (g)	B, pH	C, *C* (mg/L)	Adsorption rate of F^−^ (%)
1	0.06	5.0	150	62.8
2	0.06	6.0	60	81.2
3	0.06	7.0	100	73.9
4	0.08	5.0	100	86.8
5	0.08	6.0	150	75.1
6	0.08	7.0	60	48.9
7	0.1	5.0	60	80.4
8	0.1	6.0	100	91.8
9	0.1	7.0	150	84.6
*K*_1_	217	230	222	—
*K*_2_	210	247	211	—
*K*_3_	255	207	251	—
*k*_1_	72.6	76.7	74.1	—
*k*_2_	70.3	82.3	70.2	—
*k*_3_	85.2	69.1	83.8	—
*R*	14.9	5.65	13.6	—

## 4 Conclusion

This study focused on the mechanism of fluorine adsorption by the diatomite (DA) and biochar adsorbents. The characterization of four adsorbent materials was investigated by FEI-SEM, XRD, and FT-IR. The DA material has an excellent surface structure and is rich in oxygen-containing functional groups vis-à-vis the other three tested materials, conferring to it a strong adsorption force for F^‐^. The adsorption of DA for F^‐^ better conforms to the Freundlich model, indicating that the process entails adsorption–complex electron interaction, surface adsorption, and multi-molecular layer adsorption, while the adsorption process of CB or BC for F^‐^ occurs mainly *via* ion exchange with the participation of positive and negative charges, involving a unimolecular layer and multi-molecular layer co-adsorption process. The adsorption process of RHB on F^‐^ follows the Langmuir model, mainly characterized by unimolecular layer adsorption with ion exchange. The quasi-second-order kinetic models fitted better to the dynamics of CB, indicating that their adsorption process was dominated by chemical electron bonding adsorption, whose adsorption rate was mainly controlled by chemisorption. By contrast, a quasi-first-order kinetic model yielded a better fit for RHB, indicating that its adsorption of F^‐^ was dominated by physical adsorption. The adsorption capacity of the four materials was D > CB > BC > RHB in order of magnitude. The adsorption rate of F^‐^ by DA was 91.8% at a fluoride mass concentration of 100 mg/L, a pH of 6.0, and a dose of 4.0 g/L. The factors influencing how DA’s adsorption of fluoride in water was, in order of magnitude, dosing > mass concentration > pH. Taken together, these results reveal that DA can be recommended as an effective adsorbent to remove fluoride from contaminated water.

## Data Availability

The raw data supporting the conclusion of this article will be made available by the authors, without undue reservation.
